# Evaluating the implementation and impact of a pharmacy technician-supported medicines administration service designed to reduce omitted doses in hospitals: a qualitative study

**DOI:** 10.1186/s12913-019-4146-6

**Published:** 2019-05-22

**Authors:** Elizabeth M. Seston, Darren M. Ashcroft, Elizabeth Lamerton, Lindsay Harper, Richard N. Keers

**Affiliations:** 1 Centre for Pharmacoepidemiology and Drug Safety, Division of Pharmacy and Optometry, School of Health Sciences, Faculty of Biology, Medicine and Health, University of Manchester, Manchester Academic Health Science Centre, University of Manchester, Oxford Road, Manchester, M13 9PT UK; 2 NIHR Greater Manchester Patient Safety Translational Research Centre, University of Manchester, Manchester, UK; 3 Salford Royal NHS Foundation Trust, Salford, UK

**Keywords:** Pharmacy technician, Patient safety, Omitted dose, Medication error, Qualitative study

## Abstract

**Background:**

Of the various types of medication administration error that occur in hospitals, dose omissions are consistently reported as among the most common. It has been suggested that greater involvement from pharmacy teams could help address this problem. A pilot service, called pharmacy TECHnician supported MEDicines administration (TECHMED), was introduced in an English NHS hospital for a four-week period in order to reduce preventable medication dose omissions. The objective of this study was to evaluate the implementation, delivery and impact of the pilot TECHMED service using qualitative methods.

**Methods:**

Semi-structured interviews with pharmacy technicians, nursing staff and senior management involved with the pilot service were undertaken to evaluate TECHMED. Interviews were transcribed verbatim and analysed using the framework approach, guided by Weiss’s Theory Based Evaluation model.

**Results:**

Twenty-two stakeholder interviews were conducted with 10 ward-based pharmacy technicians, nine nurses and three members of senior management. Most technicians performed a range of activities in line with the service specification, including locating drugs from a variety of sources, and identified situations where they had prevented missing doses. Nurses reported positive impacts of TECHMED on workload. However, not all technicians fully adhered to the service specification in regard to directly following nursing staff during each medication round, citing reasons related to productivity or perceived intrusiveness towards nursing staff. Some participants also reported a perceived lack of impact of TECHMED on medicine omissions. Seventeen of the 22 interviewees supported an extension of the service. There were however, concerns about the impact on technician workload and some participants advocated support for targeted service extension to wards/rounds with high schedule dose volumes and omitted dose rates.

**Conclusions:**

The findings of this study suggest that the implementation of a pharmacy technician-supported medicines administration scheme to reduce omitted doses may be acceptable to staff in an NHS hospital, and that issues with service fidelity, staff resource/capacity and perceived interventions to avoid dose omissions have important implications for the feasibility of extending the service. The study has identified targets for future development in relation to individual and system factors to improve operationalisation of technician-led initiatives to reduce medicines omissions.

**Electronic supplementary material:**

The online version of this article (10.1186/s12913-019-4146-6) contains supplementary material, which is available to authorized users.

## Background

Globally, medication dose omissions are widely recognised as among the most common types of medication administration error that occur in hospitals [1–4]. Prescriptions not signed to record administration, patient refusal to take the dose or drug not being available on the ward are frequently among the three most common types of omitted medication dose observed in hospital studies worldwide, and, with the possible exception of patient refusals, these could be considered to be ‘preventable’ in nature [5–9].

Whilst a number of different interventions have been suggested to reduce the burden of omitted doses in hospitals [10], pharmacy team supported medicines administration activities have received recent attention for both omitted doses [11] and to improve other aspects of medicines administration quality [12]. However, there is limited attention paid to these interventions in terms of how they are perceived and implemented, and how these factors in turn might influence their impact in clinical practice. Pharmacy supported medicines administration services are complex as they require different active and interacting components that connect with the changing health care landscape to produce effects, and evaluations must therefore acknowledge the input and perspectives of different components in order to fully understand the services’ impact [13]. By seeking to explicitly explore these issues, qualitative evaluations of these complex services may better inform clinical practice and policy by understanding more clearly their implementation, why and how they produce their effects, which factors underpin their apparent success or failure, and how (and if) they might be optimised and disseminated more widely in the future [14].

In order to explore the feasibility and impact of introducing trained pharmacy technicians on medication administration rounds to accompany nurses and help promote timely medicines administration to reduce omitted doses, a pilot service was introduced by a National Health Service (NHS) hospital in the North West of England. Called pharmacy TECHnician supported MEDicines administration (TECHMED), the service was provided during a four-week period between February–March 2016.

An in-depth qualitative evaluation was undertaken with the aim of exploring the implementation, delivery and impact of the TECHMED service, and identifying individual and organisational factors to guide future service optimisation.

## Methods

### The study hospital

The study site was large university teaching hospital in the North West of England with over 750 acute medical beds on site and nearly 7000 staff, providing a comprehensive range of services to a population of approximately 250,000. Wards were selected to receive TECHMED that covered both medical and surgical specialities, had similar arrangements for medication round times and had similar patient bed numbers. The three wards receiving TECHMED were a male surgical ward, a mixed gastro-medical ward and a mixed ageing and complex medicine (elderly care) ward, each with capacity for approximately 25 patients.

On the wards receiving TECHMED services, nurses (+/− trainee nurses) conducted four routine medication rounds to administer prescribed medications; a morning round (6.30-8 am), a lunchtime round (12-2 pm), an early evening (“teatime”) round (5-6 pm) and a late evening (“bedtime”) round (9-10 pm). The standard protocol at the hospital was for nurses to make every effort to locate the drug from an alternative source where possible, or order from pharmacy if unavailable. However, baseline audit data suggested that, in practice, there were occasions where doses were omitted due to lack of supply. Traditionally, the technicians were not working on the ward when rounds were being made, so they were not in a position to assist nurses to locate drugs that were not immediately available.

In the United Kingdom (UK), pharmacy technicians work alongside pharmacists in a variety of settings in community and hospitals. Hospital pharmacy technicians may be based in the pharmacy dispensary and are traditionally tasked with the preparation and supply of medicines [15]. Pharmacy technicians are also becoming increasingly involved in ward-based activities in the UK, including medicines reconciliation, patient counselling, and supply functions [15, 16].

As part of their routine work, ward-based pharmacy technicians at the study hospital were generally assigned to up to three wards which they would visit on a regular basis to check stocks of medication, answer medicines related queries, perform medicines reconciliations with newly-admitted patients and help prepare and source medication for hospital discharge. Technicians would also spend time in the dispensary, assisting with labelling and accuracy checking of medications and answering medicines related queries.

### The TECHMED service

The inclusion criteria for technicians to participate in the service were willingness to deliver the service and the capability to alter their routine working duties and/or shift patterns. Ten ward-based pharmacy technicians employed by the host organisation volunteered to provide the TECHMED service. The aim of TECHMED was to ensure that existing prescribed drug treatment was delivered in a timely manner, and omitted doses were minimised. The hospital management and administration were supportive of TECHMED, with senior nursing and pharmacy management supporting the pilot intervention at a strategic level within the hospital. Senior management also participated in the interviews.

Participants received training on the TECHMED service in the form of a once-only face-to-face session for all participants. The training session was an interactive seminar containing a justification for the service, an outline of the TECHMED service model and what was expected of technicians delivering it. It also included case studies to apply learning and explore how TECHMED would be delivered in practice. Ward nurse managers received the same training, but without the case studies. Ward managers also distributed written and verbal information about the service to their ward staff.

TECHMED involved pharmacy technicians accompanying nursing staff on three medication administration rounds (excluding 9-10 pm round) on 5 weekdays during a 4-week delivery period. The 4-week pilot service period was chosen due to local Trust capacity and capability reasons. The TECHMED service was not provided at weekends as pharmacy technicians worked weekends on a rota based system and the department was not fully staffed. As a result, the focus was on providing dispensary-based services, and ward-based work was not routinely undertaken at weekends on all wards.

Due to variations in the working hours of pharmacy technicians who agreed to deliver TECHMED, six technicians were assigned to work on ward 1, two assigned to ward 2 and five to ward 3. Medication administration rounds happen four times a day at the study hospital; at breakfast, lunchtime, 5 pm and bedtime. During the medication round nurses administer prescribed medicines to patients. Multiple nurses on the ward complete each medication round simultaneously, with each nurse working with a different group of patients.

During the medicines administration rounds, technicians were expected to directly accompany and support a nurse on their medication round, while also being available to other nurses on the ward should they need assistance during their own medication rounds. They were advised to accompany different nurses on each medication round where possible (i.e. to not follow the same nurse at subsequent rounds). Duties expected of TECHMED technicians were:

- finding medicines currently unavailable on the ward from alternative sources (e.g. from pharmacy, other wards, etc.),
- assisting with locating medicines on the ward ready for timely administration,
- supporting nursing staff in accurate and timely documentation of medicines administration and dose omission, and
- working with nurses to liaise with ward pharmacists and medical staff when patients refuse to take doses, particularly if these involved “critical list” medicines, including anti-infectives, anticoagulants, insulin and medicines for Parkinson’s disease [17].

Dose omissions were recorded in the electronic prescribing and medication administration system. Dose omissions may still have occurred as not all omissions are due to missing medication, e.g. patient refusal, clinical reasons or patient asleep. In the case of patient refusal to take the dose, the technicians were encouraged as part of TECHMED to be available to discuss the refusal with their pharmacist and nursing colleagues and find potential reason(s) that could be corrected, but ultimately the patient had the right to refuse a medication. Such refusals were therefore recorded in the system and were not considered to be preventable.

Where practical, technicians were assigned to deliver TECHMED services on wards they routinely worked on; each technician continued to carry out other routine ward and dispensary based duties alongside TECHMED service commitments. In order to deliver the service, technicians were offered flexible working arrangements or overtime payments.

### Qualitative interviews

All technicians delivering TECHMED (*n* = 10) were invited to participate in a one-to-one interview, as were qualified nursing staff on the three participating wards and senior members of nursing/pharmacy management.

The interviews with technicians and nurses took place when the TECHMED service had been in operation for just over 2 weeks, with the remainder of the interviews taking place over a subsequent 6 week period. The interviews with senior management all took place in the 4 weeks after the service delivery period due to scheduling issues. Interviewees were asked a series of questions about their understanding of the purpose of the service, their experiences of its introduction and delivery, perceptions of the service and its impact, and views regarding feasibility and improvement. The interview schedule was developed for this study and was the same for all participants (see Additional file 1 for a summary interview schedule). The interviews were digitally audio-recorded and transcribed verbatim.

### Data analysis

The qualitative interview transcripts were imported into NVIVO 10 (QSR)® for coding and thematic analysis was undertaken by EMS using the Framework approach [18], with the thematic framework guided by Weiss’s Theory Based Evaluation model (see Table 1 for a summary of this model) [19]. RNK independently coded 11 interview transcripts using the coding framework to confirm accuracy. RNK and EMS then met to resolve any differences.

**Table 1:** Logic of analysis in evaluation (based on Weiss [19])

1. What went on in the programme over time? *Describing*	
a. Actors	
b. Activities and services	
c. Conditions of operation	
d. Participants’ interpretation	
2. How closely did the programme follow its original plan? *Comparing*	
3. What characteristics are associated with success of the programme? *Disaggregating*	
4. What combinations of actors, services and conditions are associated with success and failure? *Profiling*	
5. What recommendations do the findings imply for modifications in programme and policy? *Fashioning recommendations*	

Weiss’s model explores what happens within a particular intervention over time, describing who was involved, what activities or services were provided, how the intervention operated and participants’ interpretation of events [19]. Use of this model facilitates an understanding of how closely the delivery of TECHMED followed its original plan (fidelity) and the identification of characteristics associated with success or failure.

In addition to interview participation, pharmacy technicians delivering TECHMED services recorded data for each medication round they supported, including the ward, medication round (e.g. morning), time taken to complete the round and years of experience and seniority (denoted by NHS Agenda for Change (AfC) pay scale) of the nurse they were following. Medication round timing data was analysed in SPSS v.22 (IBM)®, using Independent Samples T-tests with the significance level set at 5%. Standard deviations (SD) are also reported for mean values. This data was used to complement and support the qualitative analysis.

## Results

Twenty-two stakeholder interviews with all 10 pharmacy technicians who delivered TECHMED, nine members of nursing staff and all three members of senior nursing/pharmacy management involved in planning/delivering the service were conducted. The mean interview time was 22 min (SD ± 8.8).

### Describing the TECHMED intervention

#### What went on in the TECHMED service over time?

All but one of the interviewees was female. Technicians had been qualified for a mean of 17 years (SD ± 14.3); nurses had been qualified for a mean of 6 years (SD ± 6.4). There were a total of 180 possible ward rounds during the TECHMED service and data were collected on 178 of the 180 rounds. Pharmacy technicians supported a median of 12 rounds (inter-quartile range = 6.5–21.25) with four of the 10 technicians providing support to all three medication rounds covered by TECHMED (morning, lunchtime and early evening) and three technicians supporting more than one ward.

#### Activities and services

Participants’ provided a number of examples of how TECHMED functioned, which often involved collaborative working, sourcing medicines from different locations and ensuring administration of doses that required prompt administration, such as antibiotics and anti-epileptic drugs.

Technicians reported that they chose one ward bay to support per medication round (each bay contained a group of patient beds), choosing a new bay on each round where possible. The number of patients in each bay (collection of patient beds) varied, depending on the ward and whether the bay included side rooms. Technicians also ensured that nurses on other bays were aware they were available to help. This activity aligned well with the original service specification.

Technicians used their knowledge of the hospital electronic dispensing database to identify medication locations across the hospital, visiting nearby wards or local automated dispensing cabinet rooms to source doses. The technicians also unpacked pharmacy deliveries and assisted clinical staff in identifying alternative drug formulations. If the drug was genuinely not available elsewhere in the hospital, technicians ordered it from the pharmacy dispensary.

Technicians also described training or educating nurses in finding drugs using the hospital computer system.

*“I’d say quite a lot of training for education showing nurses, do you know how to find that drug on the system, do you know how to get the ward stock list up and minimise it in turn so you can just dip in and out to see?”* [Technician, Identification number (ID)06]

In some cases, the technicians assumed more of a lead role compared to nursing staff in relation to the sourcing of missing doses during the TECHMED pilot, finding medication for the nurse, or, if it was unavailable on the ward, souring it from elsewhere.

*“If I haven’t had any meds they’ve gone and found them for me, or if they can’t find them they’ve gone and got them sourced from anywhere else.”* [Nurse, ID04]

All of the nurses who participated in the service (*n* = 8), described the impact of TECHMED on their workload in a positive way, as the medication round took less time to complete because they were not being interrupted to look for missing medication doses.

*“To be honest it’s just a lot quicker when you’re doing the medication round and the tech’s actually going checking for you in other cupboards* [to find medicines]...*because you can just carry on doing the medications then come back to that tablet last.”* [Nurse, ID08]

Pharmacy technicians and nurses provided a number of examples of how TECHMED had improved medicines supply on the ward, which often involved collaborative working across professional boundaries and sourcing medicines from diverse locations. The technicians reported situations in which they located missing doses, finding a different formulation when the prescribed formulation was not available, checking for unpacked drugs in the clinic room, finding drugs from other wards and putting in urgent orders for drugs that were not available elsewhere in the hospital. One technician also reported prevented a missing dose of an antibiotic, by locating it in the ward fridge.

*“I prompted the staff to look in the fridge for Co Amoxiclav liquid, because they were going to mark that as, drug not available. They didn’t know to look in the fridge for it.”* [Technician , ID07]

On another occasion the technician was able to locate a dose of an antibiotic, ceftriaxone, which s/he felt would most likely have been missed, as this was not regularly stocked on most wards.

*“It was a ceftriaxone dose that had come up and I’d caught it on the night time TECHMED, and I had a spare one in my drawer…that one could have been missed because it’s not something that’s stocked everywhere, ceftriaxone.”* [Technician, ID02]

#### Participants’ interpretation

All participants recognised the importance of missed medication doses and demonstrated good understanding of the concept of TECHMED. Three participating technicians explicitly stated that they regarded missed doses as an issue of personal priority and felt that TECHMED brought structure to this process. Senior management reported that they considered TECHMED a means of providing development opportunities for technicians and to enhance technicians’ role within the healthcare team.

There were contrasting views from technicians regarding the impact of TECHMED in regard to which professional group (technician or nurse) had responsibility for locating medication. Some technicians felt that TECHMED helped to empower nurses to locate medication, while others like this technician, felt that the responsibility for locating medication had been placed on the technicians.

*“I don’t think we’ve educated them* [nurses] *into finding it themselves….I think that some of them have got a bit lazy…they don’t have to think for themselves now because I’ll go and find everything for them.”* [Technician, ID02]

### How closely did TECHMED follow its original service specification?

Accounts from some nurse and technician participants interviewed indicated that adherence to the service specification was good, with technicians letting nursing staff know they were available to provide support and choosing different bays to support on each round. However, accounts from five of the 10 participating technicians revealed that they were not always adherent to the direction to directly follow the nursing staff during each round, in order to be present to provide support. The rationale behind these deviations fell into two main categories; (i) issues of productivity and workload and/or (ii) perceived intrusiveness toward nursing staff. None of the nursing staff interviewed reported uncomfortable feelings or any negative impact associated with having technicians on their medication rounds.

One technician described how they did not feel that it was productive to follow the nurse for the entire lunchtime medication round as the majority of medicines administered were analgesics kept as stock on the ward. This explanation for the lack of service fidelity was also echoed by several other participants, including one who stated “*there was no point me standing behind a nurse to watch her give* [analgesic name].” [Technician, ID01].

Two technicians described feeling that their presence on the ward was intrusive or disruptive towards nursing staff. One technician reported how they chose to sit at a ward computer to complete other duties and inform nursing staff they were there for support as they felt that they were causing a distraction. One technician also expressed concern that ‘standing over’ nurses could precipitate errors, particularly among newly qualified staff.

Following receipt of feedback suggesting that some nursing staff felt the presence of technicians was obstructing nurses’ contact with patients, another technician chose to position themselves close enough to the nurse so she *“…could hear what they were saying…”* [Technician, ID08] and could therefore be aware of concerns relating to missed doses*.*

### What characteristics are associated with the success or failure of TECHMED?

A factor perceived to affect the success of technician’s ability to identify missing doses was the timing of the ward round, with the morning medication round the most effective for identifying missed doses. This was in part due to the high volume of medicines prescribed at this time of day and also due to patients arriving on the ward overnight, without their own medication.

Similarly, the early evening (“teatime”) medication round was also identified by participants as an effective round to cover in terms of identifying missed doses, as doctors often performed ward rounds in the afternoons, prescribing new medications for patients. The lunchtime round was not regarded as particularly effective for identifying missed doses, as fewer medicines were given on this round, or were available as ward stock.

Another factor affecting the success of TECHMED was the impact of the lunchtime medication round on technician workload. All of the technicians who took part in TECHMED had agreed to take overtime (or work flexibly) in order to minimise impact on routine activities and ensure their presence on morning or evening medication rounds taking place outside their usual working hours. The lunchtime medication round complicated this working arrangement, as technicians had to fit the round into their existing workload.

*“I’ve done the lunchtime one, which, if I’m honest, has been a bit more difficult than the morning and afternoon one because its right in the middle of the day, and my workload as a ward tech hasn’t been reduced in any way.”* [Technician, ID01]

Similarly, two technicians reported difficulty incorporating TECHMED duties with routine tasks on Mondays due to a heavy existing workload which included completing medicines reconciliations for patients admitted over the weekend. No technician reported missing a medication round due to pre-existing work commitments however.

*“It was more difficult for me at the time because my day to day work wasn’t any less than it would normally be so trying to fit in…Mondays and Tuesdays were particularly bad for me but just that’s because of the ward combination that I would have.”* [Technician, ID01]

Medication round timing data (*n* = 178) and participants’ accounts indicated that technicians working on a familiar ward recorded significantly shorter mean medication round times than those working on an unfamiliar ward (29 min (SD ± 13.2 min) vs. 36 min (SD ± 17.1 min), t = − 2.909, *p* < 0.05). One nurse suggested that the impact of working on an unfamiliar ward was that it could take longer because technicians would not know where to find medication stocks.

*“Being on another* [unfamiliar] *ward does make it more difficult…only because there’s a lot of different hiding places* [for medication]*, especially in the clinic. When you’re on your own ward, you know where everything is... on a different ward... it does take a lot longer.”* [Nurse, ID06]

Three of the technicians reported that delivering TECHMED on their routine ward enabled them to better manage workload by pre-empting issues, identifying new patients requiring medicines supply or starting their work earlier.

Wards with overnight admissions were identified by participants as amenable to TECHMED support, as the presence of the technician on the morning round meant that patients were not missing doses of their medications.

*“So for us it’s worked because we do get a lot of admissions in the night, whether they are from other wards or from A&E or the emergency wards… a lot of* patients *won’t have their medications when they come to us …therefore they might miss them up till dinnertime. But now having the techs around it means they can pick that up…”* [Nurse, ID07]

Medication round timing data on TECHMED wards indicated that the round took longer to complete if the technician was accompanying a junior (NHS AfC Band 5) nurse rather than a senior nurse (NHS AfC Band 6/7); 35 min (SD ± 17 min) vs. 24 min (SD ± 11 min) respectively, t = 4.180, *p* < 0.05). The overall mean time for a medication round was 33 min (SD ± 16 min). Technicians reported variation in the knowledge and awareness of missing doses amongst nurses, which could lead to variation in the length of the medication round.

### What improvement recommendations do the findings imply for TECHMED?

The majority of technicians (*n* = 7) and nurses (n = 7) who delivered TECHMED had a positive attitude to the service and extension of it, perceiving that the service had reduced the number of missed doses on the affected wards, facilitated hospital discharge and improved patient care.

*“For patient safety regards to medications…it* [TECHMED] *helps and it can only help even more in the future. So it would be fantastic if it was brought in permanently.”* [Nurse, ID07]

Despite this, there were some who had concerns about how they would manage to provide the TECHMED service unless their existing workload was not modified to compensate:

*“It would be* [more feasible] *if your workload could be reduced so that you could fit* [TECHMED] *in*…*because I’ve still got three wards and everything else to try and fit in …some days I felt like I was catching my tail all the time.”* [Technician, ID01]

The seven technicians who were supportive of TECHMED service extension felt that additional staff would be required covering more wards for this to occur, with the possibility that technicians would need to work 12 h shifts in a similar way to nurses so that they could provide more effective support on the medication rounds in the early morning and late evening.

In contrast, three technicians and one nurse were unsure about the value and/or impact of the service, with the technicians reporting a limited number of missed doses they identified and prevented while delivering TECHMED and the nurse that they did not witness any missed dose intervention from a technician during their medication rounds.

*“I do think it is really good and we are helping but I really don’t know if it’s worth the time for the amount of interventions* [to reduce missed doses] *that I feel like I’ve made.”* [Technician, ID05]

Instead of a wholesale extension of the TECHMED service in its current form across the hospital, some technicians and senior managers suggested that the service could be targeted to particular wards (e.g. high numbers of agency staff, new admissions), or particular medication rounds (e.g. where most medications were administered/interventions made).

*“I think we need to look and see where did technicians make the most interventions? If there’s a particular shift that seemed to make more interventions, those you would concentrate on.”* [Senior management, ID02]

## Discussion

The findings of this study suggest that the implementation of a pharmacy technician-supported medicines administration scheme to reduce omitted doses may be acceptable to staff in an NHS hospital, but issues with service fidelity, staff resource/capacity and perceived interventions to avoid dose omissions have important implications for the feasibility of extending the service. The pharmacy technician and nurse participants broadly welcomed the service as an opportunity to work together to improve the supply of medicines. There was evidence to suggest that TECHMED had led to situations where missed doses were avoided and patient care improved by the technicians’ participation on the medication round. However, the potential impact of TECHMED on reducing omitted doses may not have been realised as some technician and nurse participants felt that they had only made a limited number of interventions to prevent missed doses during service delivery. Our findings indicate that stakeholders may not have been exposed to missed dose opportunities due to issues relating to ward familiarity, workload and TECHMED service fidelity that were identified by participants. The lack of service fidelity concerned some pharmacy technicians who reported that they did not always directly follow the nurse on the medication round due to reasons including perceived intrusiveness (including medication error risk) and workload (e.g. lunchtime medication round). These technicians could be seen to have participated in the TECHMED service, but had chosen to provide the service in a way that they thought was appropriate. Although these pharmacy technicians reported alternative methods of supporting nursing staff which they felt enabled them to respond promptly to nurse medication requests, it remains a possibility that this change could have adversely impacted on opportunities to rectify missed doses.

Globally, the number of pharmacy technicians has been growing over the past 20 years and their roles may have changed, particularly in the hospital setting [20, 21]. In the United States of America there is support for advancing use of pharmacy technicians and developing opportunities for ‘technician specialization’ [22]. Recent recommendations designed to improve operational productivity and performance in English hospitals by Lord Carter were that “*clinical pharmacy technicians* [should] *spend more time on patient-facing medicines optimisation activities*” [23]. Recent surveys of pharmacy technicians from the UK and USA also indicate that many technicians support extending their roles beyond traditional duties across hospital and community care settings respectively, often with an emphasis on clinical care [15, 24].

Our study provides novel insights into how such extended, patient facing clinical roles for pharmacy technicians may be implemented in the NHS and our findings have important implications for the design of future pharmacy technician-led medicines administration services, which may include medication error/awareness management training and ensuring that nurses and technicians meet early to discuss expectations and concerns with service delivery. Future research to illuminate the dynamic between nursing staff and technicians in delivering TECHMED may also be helpful, in order to explore further the nature and influencing factors behind service fidelity as well as where omitted dose interventions can be/are made during the medication round. For example, observational research methods have previously been used to good effect in pharmacy settings [25, 26] and may be of benefit in achieving this goal.

In this study, a number of factors were identified that influenced the success or otherwise of TECHMED and should be important considerations for future service development. These included technician familiarity with the ward they were providing TECHMED on which could have influenced their ability to locate medicines, as well as the morning and evening medication rounds which presented more opportunities to identify and rectify missed doses due to patient admissions and the volume of prescribed doses at these times of day. Workload was also implicated with regards to releasing nursing ‘time to care’, a potential benefit that should be evaluated quantitatively in future. For technicians, the importance of balancing delivering TECHMED against existing duties and the need to address long term flexible/altered working arrangements to ensure sustainability of the service were additional workload considerations. Adequate resourcing to provide the service needs to be considered and formally evaluated if other hospitals were to consider implementing the service, including recruiting sufficient technician numbers, and determining their regular duties and working hours. Extrapolating from the 2013 census of registered technicians in Great Britain suggests that approximately 10,000 pharmacy technicians are thought to be working in the hospital sector [27, 28], compared with 280,000 full-time equivalent nursing staff [29]. An alternative approach to TECHMED delivery suggested by our participants would be to implement a more targeted approach, focusing on certain rounds and wards where omitted dose rates are higher, for example.

Further research may be required to explore how pharmacy technicians work in different settings and health economies, as the configuration at the NHS hospital in question, where pharmacy technicians have ward-based roles, may not be replicated elsewhere [30]. There is evidence from the UK to indicate wide variation in the levels of ward-based clinical pharmacy activities [31] and there may also be considerable diversity in how pharmacy technicians support the work of pharmacists globally [20].

Strengths of this study included the use of in-depth interviews informed by an established intervention evaluation model with a variety of stakeholders involved with the implementation and delivery of the TECHMED pilot service. This helped to capture a broad range of perceptions and to explore important issues influencing its successful implementation and potential impact in clinical practice. The use of quantitative ward round data helped to enhance and support the qualitative findings, and the researcher conducting the interviews was also not a member of the hospital team or a health care professional which may have helped present a non-threatening / judgmental atmosphere for interviewees.

### Limitations of the study

There are a number of possible limitations to the study. Our findings may not be transferable to other health care settings as this was a single hospital site evaluation involving an enhanced role for specific pharmacy technician staff. The technicians who agreed to provide the TECHMED service, all of whom were interviewed as part of the study, were by nature self-selecting, as they had to have the capability of working additional shifts or flexibly in order to provide the service. It is possible therefore that they may not be fully representative of the technician population at the hospital. It could also be argued that the technicians may also have had a vested interest in providing a positive view on the TECHMED service as they had in some cases benefitted from receiving overtime payments for participating.

All the interviews were conducted by one interviewer (EMS) and as such, this could be considered a possible source of bias [32]. However, it should be noted that EMS is not a pharmacy or nursing professional and this was made clear to participants at the start of the interviews.

The service ran for 4 weeks due to local capacity issues and it may be that attitudes to the service may have been different had the service been in operation for a longer period. Participants did discuss during the interviews the fact that it would not be feasible to operate the service long-term with current work arrangements.

Both pharmacy technicians and nurses received training regarding the service. However, the nurse training was provided to ward managers only, who were tasked with cascading the information down to ward-based nursing staff. As this was outside the control of the research team, it is possible that some nurses did not share this information effectively with their team. This could have had an impact on nursing staff engagement and understanding of the service. It is possible that the nurses who came forward for interview were more positive towards the intervention than those who did not volunteer. However, the accounts from the nursing staff were consistently positive towards the service and saturation of themes was observed.

## Conclusions

This in-depth qualitative evaluation has found that the implementation and delivery of a ward-based pharmacy technician-supported medicines administration service to reduce omitted doses may acceptable to key stakeholders, including pharmacy technicians, nurses and senior management in an NHS hospital. Recommendations for optimization of the service would include a more targeted approach to delivery based on risk/need along with working collaboratively to set expectations and manage concerns between staff groups involved. The study has identified multiple targets in relation to both individual and system factors to improve technician-led initiatives to reduce medicines omissions, including understanding more completely the dynamic between technicians and nurses whilst performing the medication round.

## Additional file

Additional file 1: Summary of interview schedule (TECHMED)**.** a summary of topic areas and questions for participants during interviews (DOCX 58 kb)

## Abbreviations

AfC: Agenda for Change
EMS: Elizabeth M Seston
ID: Identification number
NHS: National Health Service
RNK: Richard N Keers
SD: Standard deviation
TECHMED: Pharmacy TECHnician supported MEDicines administration
UK: United Kingdom
