# Supplementary material for: Evaluating the implementation and impact of a pharmacy technician-supported medicines administration service designed to reduce omitted doses in hospitals: a qualitative study
Source: BMC Health Serv Res. 2019 May 22;19:325. doi: 10.1186/s12913-019-4146-6 (PMC6532198; doi:10.1186/s12913-019-4146-6)
Supplement: Supplementary file 1 — Summary of interview schedule (TECHMED). a summary of topic areas and questions for participants during interviews (DOCX 58 kb) [file 12913_2019_4146_MOESM1_ESM.docx]

**BMC Health Services Research (BHSR-D-18-01456R2)**

**‘Evaluating the implementation and impact of a pharmacy technician-supported medicines administration service designed to reduce omitted doses in hospitals: a qualitative study’**

Liz Mary Seston, PhD, MA(Econ), BA(Hons); Darren M Ashcroft, PhD; Elizabeth Lamerton; Lindsay Harper; Richard N Keers, PhD

**Additional file 1: Summary of interview schedule (TECHMED)**

***Questions/topics included:***

- Biographical information about the interviewee (time in post, grade, time at study hospital)
- Understanding of the purpose of TECHMED
- Awareness of the service prior to it starting
- Were participants provided with sufficient training to provide the information?

***Experience of the intervention***

- Delivery of the service on the ward (what they did in practice, who they worked with, wards worked on, etc.)
- Impact of TECHMED service on: working patterns/workload; time spent on particular activities; length of ward round; relationships with team/other health professionals; management support; workflow; communication; patient care
- Example of when medication administration successfully improved by TECHMED
- Example where TECHMED struggled to improved mediation administration

***Attitudes to the intervention***

- Did participants perceive any positive benefits to the TECHMED service?
- Were there things that did not work well, and if so, what were these?
- Identification of problems at early stage of the service, were these overcome?
- Overall attitude to service – should it be extended, and if so, how?

***Post intervention***

- What has changed, if anything since the service was removed from the wards?
- Has the service had an impact on behaviour or participants’ work practices (for pharmacy technician and nursing staff)?
- Do participants have any suggestions as to how to refine TECHMED, were it to be introduced elsewhere in the hospital?
